# DNA methylation modification in Idiopathic pulmonary fibrosis

**DOI:** 10.3389/fcell.2024.1416325

**Published:** 2024-06-10

**Authors:** Lu Ren, Yan-Fen Chang, Shi-He Jiang, Xiao-Hong Li, Hai-Peng Cheng

**Affiliations:** ^1^ Clinical Nursing Teaching and Research Section, Department of Dermatology, The Second Xiangya Hospital, Central South University, Changsha, China; ^2^ Medicine School, Zhengzhou University of Industrial Technology, Zhengzhou, China; ^3^ Department of Pathology, The Second Xiangya Hospital, Central South University, Changsha, China

**Keywords:** Idiopathic pulmonary fibrosis, DNA methylation, demethylation, methyltransferase, ten-eleven-translocation protein

## Abstract

Idiopathic pulmonary fibrosis (IPF) is a chronic, progressive, and irreversible interstitial lung disease with a prognosis worse than lung cancer. It is a fatal lung disease with largely unknown etiology and pathogenesis, and no effective therapeutic drugs render its treatment largely unsuccessful. With continuous in-depth research efforts, the epigenetic mechanisms in IPF pathogenesis have been further discovered and concerned. As a widely studied mechanism of epigenetic modification, DNA methylation is primarily facilitated by DNA methyltransferases (DNMTs), resulting in the addition of a methyl group to the fifth carbon position of the cytosine base, leading to the formation of 5-methylcytosine (5-mC). Dysregulation of DNA methylation is intricately associated with the advancement of respiratory disorders. Recently, the role of DNA methylation in IPF pathogenesis has also received considerable attention. DNA methylation patterns include methylation modification and demethylation modification and regulate a range of essential biological functions through gene expression regulation. The Ten-Eleven-Translocation (TET) family of DNA dioxygenases is crucial in facilitating active DNA demethylation through the enzymatic conversion of the modified genomic base 5-mC to 5-hydroxymethylcytosine (5-hmC). TET2, a member of TET proteins, is involved in lung inflammation, and its protein expression is downregulated in the lungs and alveolar epithelial type II cells of IPF patients. This review summarizes the current knowledge of pathologic features and DNA methylation mechanisms of pulmonary fibrosis, focusing on the critical roles of abnormal DNA methylation patterns, DNMTs, and TET proteins in impacting IPF pathogenesis. Researching DNA methylation will enchance comprehension of the fundamental mechanisms involved in IPF pathology and provide novel diagnostic biomarkers and therapeutic targets for pulmonary fibrosis based on the studies involving epigenetic mechanisms.

## 1 Introduction

Idiopathic pulmonary fibrosis (IPF) is one of the most common interstitial lung diseases, with elevated morbidity rates and limited efficacious pharmacotherapies, eventually leading to respiratory failure and death (Spagnolo et al., 2021). Its pathological changes include a damaged alveolar network progressively replaced by fibrotic scars, numerous proliferated fibroblasts continuously differentiated into myofibroblasts, and excessively deposited extracellular matrix (ECM), which in turn causes lung tissue deformation and scar formation, and eventually causes lung structure and function destruction until organ failure (Stella and Balestro, 2015). IPF is an age-related disorder, and its economic burden is expected to increase steadily with the population aging worldwide (Spagnolo et al., 2021). In recent years, the mortality of IPF has been earlier, with a median survival period of 2–4 years after diagnosis, leading to reduced quality of life (Sharif, 2017). Although there are many studies on IPF, its pathogenesis is still elusive, and the current treatment methods are also limited, mainly including lung transplantation, pulmonary rehabilitation, oxygen therapy, and monotherapy with pirfenidone and nintedanib (Raghu et al., 2011; Saito et al., 2019). Among the treatment options, lung transplantation stands as the sole efficacious intervention for IPF patients, but its practicability is poor due to the limited cost, donor, and comorbidities (George et al., 2019). Pirfenidone and nintedanib have been approved for the treatment of IPF. Nevertheless, their effectiveness in halting disease advancement and enhancing life quality remains limited, and they are also associated with tolerability concerns (Saito et al., 2019). These studies demonstrated the critical importance of drug discovery efforts and theoretical investigations into underlying mechanisms.

Epigenetics pertains to heritable alterations in gene expression and regulation that transpire without modifying the DNA sequence. Specifically, mechanisms such as DNA methylation, histone modification, modulation of chromatin architecture and dynamics, as well as the involvement of long noncoding RNAs, collectively govern the accessibility of genetic material to the transcriptional machinery and contribute to post-transcriptional control of protein translation (Velagacherla et al., 2022). This review summarizes DNA methylation of epigenetics alternation in IPF disease. Among the repertoire of epigenetic modifications, DNA methylation stands as one of the extensively studied and well-characterized mechanisms and regulates various cellular processes (e.g., proliferation, differentiation) in common respiratory diseases (Duan et al., 2022). DNA methylation patterns include methylation alteration and demethylation alteration, and their dysregulation can lead to cellular dysfunction. DNA methylation mainly involves the direct chemical modification of DNA, most of which occur on cytosine before guanine nucleotide or CpG site, and it is used in the diagnosis, prediction, and prognostic prediction of some diseases (Wu and Zhang, 2010; Moore et al., 2013; Camarena and Wang, 2016). DNA methylation is intricately linked to the transcriptional suppression of associated genes, whereas DNA demethylation induces gene reactivation and expression. In recent years, with the continuous research on DNA methylation modification, studies have confirmed that it is involved in the pathogenesis of a variety of respiratory diseases, such as pulmonary fibrosis, asthma, chronic obstructive pulmonary emphysema, and lung cancer, and can cause further aggravation of these diseases (Qiu et al., 2012; Zhang et al., 2019; Chen et al., 2020; Sheikhpour et al., 2021). This review article primarily focuses on delineating the pathological features of IPF and elucidating the impact of DNA methylation alterations in its pathogenesis, then provide potential future ideas for IPF prevention and treatment.

## 2 Pathologic features of IPF

IPF is a fatal lung disease of unknown etiology, characterized by the damage of alveolar epithelial cells (AECs), the proliferation and differentiation of fibroblasts, and the formation of myofibroblast foci, accompanied by abnormal gene expression and ECM deposition (King et al., 2011). The cardinal feature of IPF is characterized by excessive fibroproliferation, with activated fibroblasts serving as the key contributors to the process of fibrogenesis (Spagnolo et al., 2021). Studies based on the prevalence of IPF have shown that histopathological analysis of fibrotic lungs commonly exposes significant alveolar scarring, wherein the normal alveolar structures are substituted with fibrous scars populated by myofibroblasts due to the abnormal healing of repetitive lung injury wounds (Selman et al., 2001). The impairment of alveolar epithelial function is hypothesized to play a pivotal role in the initiation of IPF. The damaged and aberrant AECs disrupt normal epithelial repair and propagate profibrotic phenotypes, eventually lead to the proliferation, recruitment, and activation of fibroblasts/myofibroblast, and the synthesis of ECM (Ahluwalia et al., 2014). These studies indicate that AECs damage and dysregulation play a key role in IPF pathogenesis.

Fibroblastic foci, formed mainly by fibroblasts, myofibroblasts, and ECM proteins produced by myofibroblasts, are the most typical pathological features of IPF (Kuhn and McDonald, 1991). Myofibroblasts, as effector cells, are responsible for the excessive accumulation of ECM proteins, leading to the compromised structure and function of the lung. They are recognized as the primary source of heightened deposition of ECM proteins, including fibrillar type I and III collagens, not only in the fibrotic lungs but also in other fibrotic organs (Todd et al., 2012). Activated myofibroblasts are a spindle or star state with a contractile phenotype and intracytoplasmic stress fibers, which can stimulate the transcriptional activity of collagen genes along with the elevation of mesenchymal proteins, including α-SMA and vimentin (Schürch et al., 1998). They are also key mediators of ECM, structural remodeling, and destruction of pulmonary capillaries during/after lung injury (Phan, 2002). The prognostic outlook of IPF is intricately linked to the severity of fibroblastic foci observed during pathological examination.

The proliferation and accumulation of resident fibroblasts represent crucial processes in the pathogenesis of IPF, wherein the differentiation of fibroblasts into myofibroblasts serves as a principal source of myofibroblasts within fibroblastic foci, contributing to fibrogenesis (Fernandez and Eickelberg, 2012). Activated fibroblasts serve as the primary origin of ECM production (Spagnolo et al., 2021). Furthermore, epithelial-to-mesenchymal transition (EMT) contributes to the presence of fibroblasts derived from AECs within fibroblastic foci. EMT entails the progressive loss of epithelial markers (e.g., E-cadherin, keratin) accompanied by persistent upregulation of mesenchymal markers (e.g., α-SMA, N-cadherin, vimentin, fibronectin) (Willis et al., 2006). TGF-β1, a recognized profibrogenic mediator, is a key factor that promotes the proliferation and differentiation of fibroblasts into myofibroblasts through Wnt/β-catenin signaling and also induces AECs to undergo EMT through activation of the Ras/ERK/MAPK signaling cascade (Marmai et al., 2011; Liu et al., 2012). In summary, pathological fibrogenesis in IPF represents a dynamic process characterized by intricate interplay among various cellular components, including epithelial cells, fibroblasts, and myofibroblasts, et al.

## 3 Roles of altered DNA methylation modifications in IPF

**FIGURE 1:**
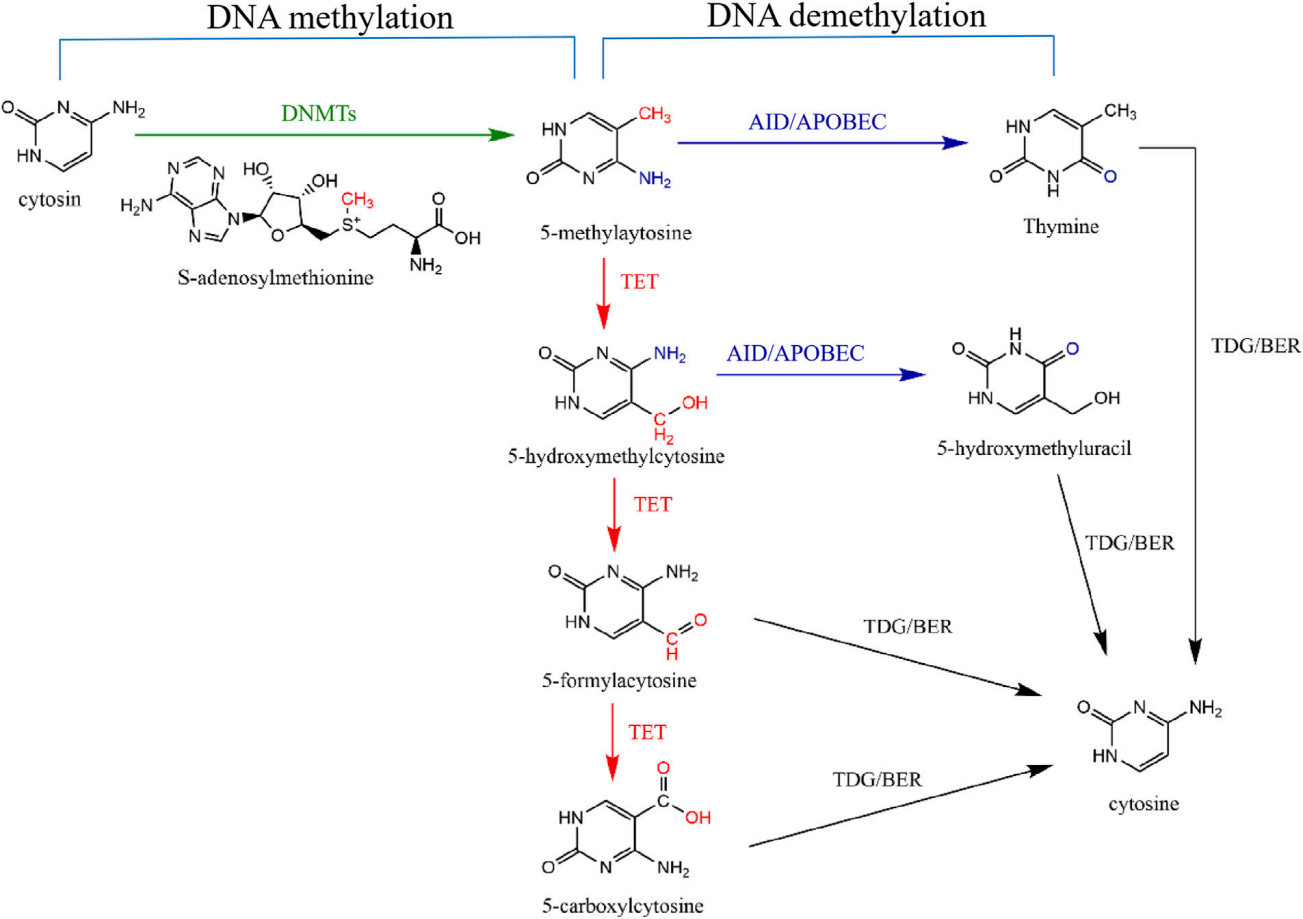
DNA methylation and demethylation pathways. DNA methylation means DNMTs transfer the methyl group on S-adenosine-L-methionine to carbon five of cytosine to form 5-methylatosine (5-mC) (green line). DNA demethylation means that 5-mC is replaced by cytosine through two signaling pathways. Active DNA demethylation means that Ten-Eleven-Translation (TET) protein first oxidizes 5-mC to produce 5-hydroxymethylcytosine (5-hmC), 5-aldehydecytosine (5-fC) and 5-carboxylcytosine (5-caC) (red lines). Subsequently, thymidine DNA glycosylase (TDG) can selectively recognize and cut the glycosidic bonds of 5-fC and 5-caC to form a base-free AP site and then start the downstream pathway of base excision repair (BER) to reduce 5-mC to unmodified cytosine (black lines). Moreover, after TET-mediated oxidation of 5-mC to 5-hmC, AID/APOBEC deaminase can deaminate 5-hmC to 5-hydroxymethyluracil (5-hmU) (purple route) and then be further excised by TDG/BER signaling pathway (black line). 5-mC can also be directly deaminated by AID/APOBEC to generate thymine (purple route), which is then converted to C by the TDG/BER signaling pathway (black line).

Recently, the significance of epigenetic mechanisms in the IPF pathogenesis has become increasingly prominent (Moss et al., 2022). Distinctly altered DNA methylation modifications are found in IPF patients (Tzouvelekis and Kaminski, 2015). DNA modification is one of the key epigenetic mechanisms and regulates various cellular processes (e.g., proliferation and differentiation), including methylation alteration and demethylation alteration (Figure 1). The imbalance of DNA methylation status can lead to cell dysfunction or cell transformation, exhibiting close associations with the advancement of fibrosis diseases, autoimmune diseases, and malignant tumors, (Duan et al., 2022). DNA methylation alteration usually occurs in the CpG dinucleotide cluster in the promoter region (called “CpG island”) and is related to the transcription inactivation of the affected genes; on the other hand, DNA demethylation alteration induces the reactivation and expression of genes (Tzouvelekis and Kaminski, 2015). Emerging evidence suggests a pivotal role for DNA methylation dysfunction in the pathogenesis of pulmonary fibrosis (Duan et al., 2022).

### 3.1 Abnormal DNA methylation patterns

There are two abnormal methylation patterns, hypermethylation, and hypomethylation. Generally, DNA methylation patterns and gene expression exhibit an inverse association. Specifically, genes characterized by DNA hypermethylation are associated with transcriptional downregulation, whereas genes displaying DNA hypomethylation are linked to transcriptional upregulation (Mattei et al., 2022). At present, research on DNA methylation in pulmonary fibrosis has focused on abnormal DNA methylation patterns of fiber genes in IPF lung tissue or fibroblasts/myofibroblasts.

#### 3.1.1 Abnormal DNA methylation pattern in IPF lung

Rabinovich et al. found a differential methylation analysis between IPF and control lung tissues revealed 625 CpG islands with distinct methylation patterns (Rabinovich et al., 2012). Among more than 14,000 genes, 870 have abnormal methylation levels, of which 53% are hypermethylated, and 47% are hypomethylated in IPF lung tissues (Sanders et al., 2012). Among 870 genes, seven genes previously reported to be involved in IPF are claudin-5 (CLDN5), haptoglobin (HP), tumor protein P53-inducible nuclear protein 1 (TP53INP1), dimethylarginine dimethylaminohydrolase 1 (DDAH1), collagen type III alpha 1 (COL3α1), matrix metalloproteinase 7 (MMP7) and cathepsin K (CTSK) and their DNA methylation patterns are consistent with gene expression in published data (Sanders et al., 2012). Among the seven genes, CLDN5 and HP are hypermethylated, while TP53INP1, DDAH1, COL3α1, MMP7, and CTSK are hypomethylated in IPF lung tissues (Sanders et al., 2012). The hypermethylation of zinc finger protein 467 (ZNF467) gene downregulate its protein expression in pulmonary fibrosis, thereby decreasing the activation of peroxisome proliferator-activated receptor gamma (PPARγ) (Sanders et al., 2012). Activating PPARγ signaling can play an antifibrotic role (Kheirollahi et al., 2019). Recently, in both IPF patients and bleomycin (Blm)-treated mice lungs, a noticeable decline in PPARγ expression was observed concurrently with PPARγ promoter hypermethylation (Wei et al., 2022). The above studies indicate that abnormal DNA methylation patterns occur in fibrotic lung tissue. Some genes are hypermethylated, while others are hypomethylated, but their interactions and dynamic balance have not been reported in research.

#### 3.1.2 Abnormal DNA methylation pattern in fibroblast/myofibroblast

Abnormal DNA methylation patterns also occur in fibrotic fibroblasts/myofibroblasts. Lee et al., 2019 found that the comparative analysis of IPF fibroblasts with the control group revealed hypomethylation in 4,251 loci spanning 1,731 genes, while 1,599 loci across 725 genes exhibited hypermethylation tendencies. Among 178 differentially expressed genes, they observed significant correlations between the mRNA expression levels of 34 genes (13 genes were downregulated, while 21 genes were upregulated) and 80 CpGs (30 CpGs showed hypermethylation, while 50 CpGs exhibited hypomethylation) between the two groups (Lee et al., 2019). Furthermore, out of the 34 genes, the gene S100A4 has been chosen as a candidate for functional validation. Notably, there is a considerable disparity in the levels of CpG methylation between IPF fibroblasts and normal controls, with the former exhibiting significantly lower levels in comparison to the latter (Lee et al., 2019).

A study aimed to analyze the DNA methylation levels of 27,568 CpG sites across the genome in order to investigate and compare the DNA methylation patterns of IPF fibroblasts with those of nonfibrotic patient controls and commercially available normal lung fibroblast cell lines show that multiple CpG sites across the genome are differentially methylated in IPF fibroblasts (Huang et al., 2014). The study has revealed that cyclin-dependent kinase inhibitor 2B (CDKN2B) and caspase recruitment domain-containing protein 10 (CARD10) are the top two genes exhibiting hypermethylation in IPF fibroblasts (Huang et al., 2014). Another study also shows that CDKN2B gene locus are hypermethylated in the fibroblasts of IPF patients, decreasing CDKN2B protein expression and contributing to IPF pathogenesis (Scruggs et al., 2018).

The occurrence of aberrant hypermethylation plays a significant role in promoting the activation of fibrotic fibroblasts and driving fibrogenesis. For instance, RASAL1 hypermethylation in fibrotic kidney, PTEN hypermethylation in fibrotic liver, and E-Cadherin hypermethylation in fibrotic lung, has been found to be particularly important in these processes (Zeisberg and Zeisberg, 2013). The low expression of Thy-1 (CD90) is related to its hypermethylation in myofibroblasts of the fibroblastic foci during pulmonary fibrosis (Sanders et al., 2008). Thy-1 is a crucial factor involved in maintaining cellular and matrix equilibrium in normal lung fibroblasts. However, it is noteworthy that Thy-1 expression is absent in IPF fibroblasts (Hagood et al., 2005; Zhang et al., 2019). The low level of prostaglandin (PG) E_2_ and the limited capacity of upregulating cyclooxygenase-2 (COX-2) are observed in lung fibroblasts obtained from IPF individuals and contribute functionally to the fibroproliferative state (Evans et al., 2016). Chromosome 8 open reading frame 4 (c8orf4), also known as thyroid cancer protein 1 (TC-1), serves as a transcriptional regulator. In fibrotic fibroblasts, it undergoes hypermethylation, leading to its downregulation, then decreasing COX-2 expression and PGE_2_ synthesis (Evans et al., 2016). The fibrotic fibroblasts also exhibit a hypermethylation pattern of the prostaglandin E receptor 2 (PTGER2) gene promoter, mediating the downregulation of PTGER2 expression and consequent PGE_2_ synthesis (Huang et al., 2010). The significantly hypermethylated promoters of secreted frizzled-related protein (SFRP) 1 and SFRP4 are significantly associated with impaired transcription and diminished expression levels in the context of pulmonary fibrosis in mice (Zhou et al., 2019). Furthermore, the reactivation of SFRP1 and SFRP4 through the application of 5-Azacytidine (5-aza) results in a reduction of β-catenin mRNA and protein expression, thus significantly alleviating PF induced by Blm (Zhou et al., 2019). The diminished expression of the proapoptotic p14 (ARF) is correlated with the presence of a hypermethylated promoter in IPF fibroblasts. Notably, these IPF fibroblasts exhibiting hypermethylation of ARF demonstrate a significant increase in resistance to apoptosis induced by staurosporine and S-nitrosoglutathione (Cisneros et al., 2012). Hypermethylation of mothers against decapentaplegic homolog 4 (Smad4) also drives pulmonary fibrosis and elevates the susceptibility to pulmonary carcinogenesis in IPF (Takenaka et al., 2009). The above studies indicate hypermethylation of certain specific genes in lung assumes crucial functions in promoting the activation of fibrotic fibroblasts and driving the process of fibrogenesis.

Aberrant hypomethylation of some selected genes also play important roles in driving fibrogenesis. Methylation of O6-methylguanine-DNA methyltransferase (MGMT) is a hypomethylated gene, and its expression is upregulated in IPF fibroblasts (Huang et al., 2014). MGMT, a DNA repair enzyme, plays a regulatory role in chromatin stability and susceptibility to apoptosis. The elevated expression of MGMT in IPF fibroblasts is posited to potentially contribute to the widely acknowledged phenomenon of heightened resistance to apoptosis (Huang et al., 2014). Fibroblasts originating from the lung exhibit a notable elevation in the expression of the transcription factor forkhead box L1 (FOXL1). The increased FOXL1 mRNA expression is found in fibroblasts of IPF lungs and shows DNA hypomethylation and super-enhancer formation (Miyashita et al., 2020). Transcriptome analysis reveals that FOXL1 can control many genes that potentiate fibroblast function, including TAZ/YAP signature genes and PDGFRα (Miyashita et al., 2020). Rabinovich et al. find that the expression of three genes serine/threonine kinase 17b (STK17B), serine/threonine kinase 3 (STK3), and histone cluster 1 H2ah (HIST1H2AH) with hypomethylated promoters is increased in IPF lungs (Lee et al., 2019). STK17B and STK3 play crucial roles in apoptosis, whereas HIST1H2AH is essential to nucleosome formation.

The above researches demonstrate that pulmonary fibrosis related genes DNA methylation patterns are altered in lung tissues or fibroblasts with IPF (Table 1). Furthermore, a noteworthy diversity in DNA methylation patterns was identified among individual cell lines associated with IPF. This observation implies that variations in DNA methylation may potentially play a role in the heterogeneity of fibroblasts among patients diagnosed with IPF (Huang et al., 2014). Notably, only a subset of differences in gene expression exhibited a directional opposition to the disparities in methylation (Lee et al., 2019). These imply that additional investigations are required to elucidate the intricate relationship between individual gene expression variations and the influence of hypermethylation or hypomethylation.

**TABLE 1:** Genes are differentially methylated and expressed in pulmonary fibrosis.

Hypermethylation/downregulation	Functions in IPF	References	Hypomethylation/upregulation	Functions in IPF	References
CLDN5	It is expressed in lung endothelium. Its hypermethylation in IPF lung tissues indicates EMT in specific cell populations	Sanders et al., 2012	TP53INP1	It is a p53-inducible cell stress response protein, a major mediator of p53 antioxidant function. It is reported to be increased in IPF.	Sanders et al., 2012
HP	Label-free plasma proteomics identifies HP-related protein as a candidate marker of IPF and dysregulation of complement and oxidative pathways	Sanders et al., 2012	DDAH1	The increased DDAH can decrease endogenous inhibitors of nitric oxide synthase (NOS) activity, which results in increased NOS in IPF.	Sanders et al., 2012
ZNF467	Its hypermethylation can result in lowered expression in IPF and decrease the activation of peroxisome proliferator-activated receptor gamma (PPARγ) in IPF lung tissues	Sanders et al., 2012	COL3α1	COL3α1 gene can encode the protein alpha 1 chain of type III collagen, which is required to form type III collagen, an extracellular matrix protein	Sanders et al., 2012
CDKN2B	Its hypermethylation promote proliferation and differentiation of fibroblasts into myofibroblasts	Scruggs et al., 2018	MMP7	MMP7 is involved in matrix remodeling and fibrogenesis	Sanders et al., 2012
CARD10	CARD10 is a scaffold protein that associates upstream G protein signals with downstream NF-κB activity	Huang et al., 2014	CTSK	Human pulmonary fibroblasts exhibit increased activity of CTSK and increased intracellular collagenolytic activity	Sanders et al., 2012
Thy-1	The low expression of Thy-1 is related to fibroblast activation and differentiation in fibrotic lesions	Sanders et al., 2008	MGMT	It may contribute to the well-recognized phenomenon of fibroblast resistance to apoptosis in IPF.	Huang et al., 2014
c8orf4 (TC-1)	TC-1 causes COX-2 expression and PGE_2_ synthesis in fibrotic fibroblasts	Evans et al., 2016	FOXL1	It potentiates fibroblast function, including TAZ/YAP signature genes and PDGFRα	Miyashita et al., 2020
PTGER2	mediating the downregulation of PTGER2 expression and consequent PGE_2_ synthesis	Huang et al., 2010	STK17B	regulation of apoptosis	Lee et al., 2019
SFRP1 and SFRP4	reactivations of SFRP1 and SFRP4 reduce β-catenin mRNA and protein expression	Zhou et al., 2019	STK3	regulation of apoptosis	Lee et al., 2019
p14 (ARF)	p14 (ARF)-hypermethylated IPF fibroblasts were significantly more resistant to apoptosis	Cisneros et al., 2012	HIST1H2AH	nucleosome formation	Lee et al., 2019
Smad4	It is a transcription factor and mediates TGF-β signaling	Takenaka et al., 2009	S100A4	S100A4 regulates cell motility, survival, differentiation, and contractility	Lee et al., 2019

### 3.2 DNA methylation in IPF

DNA methylation plays an important role in gene regulation and genomic function. DNA methylation primarily occurs through the action of DNMTs, facilitating the transfer of a methyl group from the donor S-adenosine-L-methionine to the carbon 5 position of the cytosine base, thus producing 5-mC, which mainly exists at cytosine-phosphate-guanine dinucleotide site (Borchiellini et al., 2019; Ginder and Williams, 2022; Rustad et al., 2019) (Figure 1). Therefore, DNA methylation alterations usually occur at CpG island, a CG-rich DNA region frequently situated at or in close proximity to the transcription start site of a gene (Rustad et al., 2019). Frequently, unmethylated CpG sites can be identified by specific protein binding domains, such as the CXXC domain. This recognition prevents access to the DNA methylation machinery, thereby facilitating the maintenance of the unmethylated state (Szulwach and Jin, 2014). Studies have shown that DNA methylation plays an important role in regulating myofibroblast basic biological characteristics (Hinz et al., 2012).

#### 3.2.1 DNMTs

DNMTs can establish and maintain genomic DNA methylation patterns on the cytosine of CpG dinucleotides (Métivier et al., 2008). DNMTs are divided into two categories: *de novo* enzymes DNMT3a, DNMT3b, and DNMT3L, which can establish new methylation patterns; maintenance enzymes, such as DNMT1, which can maintain methylation modification (Okano et al., 1998; Goll and Bestor, 2005; Lyko, 2018; Schmitz et al., 2019). DNMT3a and DNMT3b are implicated in the initial establishment of DNA methylation, a process that DNMT1 subsequently maintains during DNA replication through the cell cycle. DNMT3L, lacking a conserved catalytic methyltransferase domain, facilitates *de novo* methylation by DNMT3a/b through the recognition of unmethylated histone H3 lysine 4 residues (H3K4me0) (Okano et al., 1999; Moore et al., 2013). DNMT2 mainly catalyzes RNA methylation, so we will not elaborate on it here. In mammalian cells, as unmodified cytosines are incorporated into symmetrical CpG sites during replication, DNMT1 selectively recognizes the hemimethylated CpG dyad and transfers a methyl group to the unmethylated cytosine (Szulwach and Jin, 2014). Collectively, methylation of DNA itself plays a role in gene transcription by physically hindering the combination of transcription factors and genes.

Lung fibroblasts and epithelial cells have three DNMTs, DNMT1, DNMT3a, and DNMT3b (Hu et al., 2010). DNMT3a has the highest expression in both cells, and the expression of DNMTs in AECs that do not express α-SMA is usually higher than that in fibroblasts (Hu et al., 2010). Studies show that inducing DNMT overexpression in lung fibroblasts can inhibit the expression of the *ACTA* gene, thereby inhibiting the expression of α-SMA and inhibiting the differentiation of fibroblasts into myofibroblasts (Hinz et al., 2012; Hu and Phan, 2013). The *ACTA* gene is an important mechanism for modifying gene silencing on CpG islands in the case of DNA methylation, and it exhibits high levels of methylation on AT2 cells and low levels of methylation on lung fibroblasts (Hu et al., 2011; Sanders et al., 2011; Hinz et al., 2012). Sanders et al., 2012 also have shown that the expression levels of DNMT3a and DNMT3b in the lung tissues of IPF patients are significantly increased. Elevated immunohistochemical staining for DNMT3a is predominantly localized to the nucleus and is observed across various cell types. In IPF, the staining intensity is most pronounced in the hyperplastic epithelium covering fibroblastic foci; however, there is no consistent increase observed in myofibroblasts situated within the foci (Sanders et al., 2012). DNMT3b is a major DNMT in *de novo* DNA methylation, which regulates macrophage polarization (Yang et al., 2014). DNMT3b deficiency can promote IL4-and TGF-β1-induced replacement macrophage polarization *in vitro*, and enhance the pro-fibrotic macrophage polarization in the alveolar space during pulmonary fibrosis *in vivo*, thus promoting the pulmonary fibrosis process (Qin et al., 2022). Recently, the increased DNMT1/DNMT3a were also found in IPF patients and Blm-treated mice lungs (Wei et al., 2022).

These data suggest that aberrant DNMTs elevations are found in IPF, and gene expression patterns are altered, which may play a significant role in the progression of pulmonary fibrosis. Aberrant elevations in DNMTs may be partially accountable for variations in altered methylation patterns observed in specific cell types. Consequently, further comprehensive investigation is warranted to thoroughly explore the role that DNMTs play in the context of pulmonary fibrosis.

#### 3.2.2 MBD proteins

In addition, methyl-cpg binding domain (MBD) protein family, which mainly includes MBD1, MBD2, MBD4 and MECP2, read the data encoded by DNA methylation and regulate gene transcription (Du et al., 2015). MBD proteins demonstrate affinity for 5-mC and have the capacity to facilitate transcriptional repression associated with 5-methylcytosine-guanine sites. They effectively block transcription factor binding (Zeisberg and Zeisberg, 2013).

Among MBD proteins, the MBD protein family, MBD2 has been demonstrated to exhibit the highest binding affinity to methylated CpG DNA and play a role in PF pathogenesis (Wang et al., 2021). MBD2 is notably expressed at elevated levels in lung macrophages derived from individuals with IPF as well as in mice with bleomycin-induced pulmonary fibrosis (Wang et al., 2021). In this study, MBD2 exhibits selective binding to methylated CpG DNA within the promoter region of SH2-containing inositol 5′-phosphatase (Ship). This region regulates the PI3K/Akt signaling, thereby enhancing the macrophage M2 program. The absence of MBD2 in macrophages provides significant protection to mice against Ble-induced PF, accompanied by a notable decrease in the accumulation of M2 macrophages in the lung. In another study, lungs derived from patients with IPF and mice with Blm-induced PF exhibit distinctive alterations in DNA methylation. Furthermore, myofibroblasts in these conditions show overexpression of MBD2, and the depletion of MBD2 in fibroblasts or myofibroblasts confers protection to mice against Blm-induced PF. This protection is associated with a notable reduction in fibroblast differentiation (Wang et al., 2022). TGF-β1 stimulates fibroblasts to undergo widespread DNA hypermethylation, accompanied by the overexpression of MBD2 in a TβRI/Smad3-dependent manner. Notably, MBD2 selectively binds to the methylated CpG DNA within the Erdr1 promoter, leading to the repression of Erdr1 expression. This mechanism enhances TGF-β/Smad signaling, promoting the differentiation of fibroblasts into myofibroblasts and exacerbating pulmonary fibrosis (Wang et al., 2022).

MethylCPG-binding protein 2 (Mecp2) in this family is a protein responsible for interpreting information encoded by DNA methylsets, and its overexpression mainly occurs in macrophages. By enhancing the expression of interferon regulatory factor 4 (Irf4), Mecp2 plays a role in promoting M2 macrophage polarization. Consequently, the silencing of Mecp2 expression leads to a substantial reduction in M2 macrophage polarization, as it hinders the coordinated function of Irf4. Ultimately, this mechanism contributes to the alleviation of pulmonary fibrosis (Mou et al., 2022). Furthermore, Mecp2 siRNA‐loaded liposomes obviously reverse the defined pulmonary fibrosis through intratracheal administration (Mou et al., 2022).

The above studies show that MBD proteins play a role in the pathogenesis of pulmonary fibrosis, but more systematic and in-depth research is needed. At present, the main studies focus on the roles of MBD2 and Mecp2 in pulmonary fibrosis, but their mechanism of action needs to be further studied.

### 3.3 DNA demethylation in IPF

DNA demethylation in this review mainly refers to the active demethylation (Figure 1). Active demethylation of DNA is an enzymatic process, which refers to removing methyl groups from 5-mC by breaking the thecarbon-carbon bond, then producing 5-hmC, an oxidized form of 5-mC (Wu and Zhang, 2010).

Ten-Eleven-Translocation (TET) proteins, including three members TET1, TET2, and TET3, are 5-mC dioxygenases and key enzymes mediating the active demethylation of DNA (Ito et al., 2010; Bochtler et al., 2017). All TET proteins contain a conserved double-stranded β-helical domain, a cysteine-rich domain, cofactor Fe (Ⅱ), and 2-oxoglutarate (Rasmussen and Helin, 2016). In addition to TET2, both TET1 and TET3 possess a CXXC domain. This domain enables TET1 and TET3 to recognize and bind to CpG islands (Iyer et al., 2009). TET proteins can sequentially oxidize 5-mC to 5-hmC, then 5-formylcytosine (5-fC) and 5-carboxycytosine (5-caC) (He et al., 2011; Pastor et al., 2013; Lio and Rao, 2019). The thymidine DNA glycosylase (TDG) exhibits a high specificity for recognizing and excising 5-fC and 5-caC from DNA. This enzymatic activity, when coupled with the base excision repair (BER) signaling pathway, leads to the conversion of 5-mC back to an unmethylated cytosine state (Ko et al., 2015). Moreover, after TET-mediated oxidation of 5-mC to 5-hmC, AID/APOBEC deaminases can deaminate 5-hmC to 5-hydroxymethyluracil (5-hmU) and then be further excised by TDG/BER signaling pathway (Globisch et al., 2010; Guo et al., 2011). 5-mC can also be directly deaminated by AID/APOBEC to generate thymine, which is then converted to C by the TDG/BER signaling pathway (Guo et al., 2011).

TET1 is mainly expressed in embryonic stem cells, while TET2 and TET3 are more common in various differentiated tissues, among which TET2 is dominant in the nervous and hematopoietic system (Tahiliani et al., 2009; Solary et al., 2014). Tahiliani et al. found that TET1 can act on fully methylated or hemimethylated DNA (Tahiliani et al., 2009). In addition, TET1 can be used as a maintenance DNA demethylase to specifically maintain the DNA hypomethylation status of CpG islands by preventing the diffusion of methylation from the edge of methylation and does not change DNA methylation globally (Jin et al., 2014). TET2 is commonly recognized as a tumor suppressor gene located within the nucleus. It functions as a DNA modification enzyme, playing a crucial role in the process of DNA demethylation (Pan et al., 2015). Wild-type TET2 plays a crucial role in the survival and development of hematopoietic stem cells and the maintenance and differentiation of embryonic stem cells. The loss of function induced by TET2 mutation is related to DNA hypermethylation phenotype (Cimmino et al., 2017). This highlights the significance of intact TET2 function in maintaining proper DNA methylation patterns and cellular processes.

When mouse embryonic fibroblasts (MEFs) lack the TET1 gene, the reprogramming efficiency of MET is slightly increased. TET3 knockout has almost no effect on the MET reprogramming efficiency of MEFs, while TET2 knockout or TET complete knockout significantly inhibits the MET reprogramming process of MEFs (Chen et al., 2013; Hu et al., 2014). The above studies showed that compared with TET1 and TET3, TET2 plays a more important role in regulating the phenotype and function of fibroblasts. In the early stage of IPF, a series of inflammatory cells such as macrophages will move to the injury site, and T cells will be activated to secrete inflammatory factors, leading to lung inflammation, and the loss of TET2 will affect the inflammatory response (Tsiouplis et al., 2020). The TGF-β1/Smad3 signaling pathway participates in TET1-and TET2-mediated DNA methylation modification and plays a role in regulating T cell differentiation and maintaining immune homeostasis (Yang et al., 2015). Qin et al., 2021 showed that TET2 expression is decreased in the fibrotic lungs of mice, reaching the lowest point on the 14th day after Blm challenge. The decreased expression of TET2 is also shown in the lungs and AT2 cells of IPF patients, but TET2 in the alveolar epithelium is not involved in the progression of Blm-induced pulmonary fibrosis (Qin et al., 2021). The above research shows that the decrease of TET2 may contribute to the pathogenesis of PF by affecting fibroblasts’ function instead of the alveolar epithelium, but it needs further research.

A recent study demonstrates that excessive growth differentiation factor 7 (GDF7) protein promotes the expression of pro-fibrotic genes (α-SMA and fibronectin) through the bone morphogenetic protein receptor type 2 (BMPR2)/Smad signaling (Wan et al., 2021). TET-dependent GDF7 hypomethylation increases GDF7 expression and can serve as a potential therapeutic target in glaucoma (Wan et al., 2021). Therefore, TET-mediated the change of DNA methylation pattern to regulate gene expression, which may also become a potential therapeutic target for pulmonary fibrosis. Moreover, multiple studies have shown that the enzymes of DNMT and TET protein are expressed concurrently when regulating DNA methylation, and the competition between DNMT and TET protein can realize the dynamic regulation of DNA methylation to avoid abnormal methylation (Tsagaratou et al., 2017; Tsiouplis et al., 2020).

### 3.4 Therapeutic strategies related to DNA methylation in IPF

Based on the basic research on DNA methylation patterns in pulmonary fibrosis mentioned above, researchers are also constantly exploring relevant treatment strategies. 5-azacytidine/5-aza-2′-deoxycytidine (5-aza/5-aza-dc), glycyrrhizic acid (GA), and zebularine are DNA methylation inhibitors which act as potent DNA demethylating agents, and their use in experimental model of pulmonary fibrosis has been widely concerned. 5-aza-dc and GA led to the demethylation of the PPARγ promoter, thereby restoring PPARγ levels and mitigating fibrotic lung pathologies (Wei et al., 2022). In cultured lung fibroblasts and AECs, the application of GA mitigated the PPARγ-mediated inhibition of fibrosis through a mechanism sensitive to DNMT gain (Wei et al., 2022). Dakhlallah et al., 2013 reported that in a murine Blm-induced PF model, treatment with 5-aza-dc resulted in decreased expression of fibrotic genes and DNMT1, elevated expression of the miR-17–92 cluster, and alleviation of fibrosis. 5-aza-dc also alleviated hyperoxia-induced PF in neonatal rats by downregulating TGF-β1 expression and upregulating p16 expression through reversal of hypermethylation of p16 (Zhao et al., 2018). Other studies have shown that treatment with 5-aza or zebularine and DNA methyltransferase specific siRNA can restore Ep2 mRNA transcription. Then, the response of PGE_2_ to fibroblasts was restored to maintain the homeostasis of fibroblasts and alleviate the pathological condition of pulmonary fibrosis (Tirelli et al., 2022). 5-aza-dc also reversed the inhibition of Thy-1 expression in fibroblasts induced by hypoxia, and was accompanied by a decline in α-SMA expression (Robinson et al., 2012). 5-aza-dc reduced c8orf4 methylation, restored COX-2 expression, and normalized fibroblast function (Evans et al., 2016). In fibroblasts, 5-aza had the capacity to diminish BMPER expression, thereby reducing the proliferation and migration of fibroblasts associated with IPF (Huan et al., 2015). Furthermore, treatment with 5-aza had an additional impact on modulating BMPER expression and resulted in a reduction of pulmonary fibrosis in mice (Huan et al., 2015). In a word, a group of fibrosis-related genes, including TGF-β1, CXCL10, COX-2, and Thy-1, may be potential targets for epigenetic therapy of pulmonary fibrosis. At present, the treatment of DNA methylation mainly focuses on DNA demethylation agents, and other treatment methods remain to be explored.

5-azacytidine (azacitidine, Vidaza^®^) and its deoxy derivative, 5-aza-2′-deoxycytidine (decitabine, Dacogen^®^), are the most widely used inhibitors of DNA methylation for cancer therapy which trigger hypomethylation leading to a consecutive reactivation and upregulation of epigenetically silenced tumor suppressor (Christman, 2002). Since the two demethylating agents were first approved by the U.S. FDA for the treatment of myelodysplastic syndrome (MDS) in 2004, DNA demethylating agents have sparked a widespread trend of application and been used to treat blood cancers as well as solid tumors in clinical practice (Howell et al., 2010). It is noteworthy that treatment with hypomethylating agent can lead to demethylation and upregulation of a oncogenic gene, resulting in 50% of MDS patients not responding to these drugs (Liu et al., 2022). It means that demethylating drug can upregulate the expression of oncogene while demethylating tumor suppressor gene, which not only treats tumor but also poses a high risk of carcinogenesis. Therefore, these druges may have a potential for driving fibrosis by upregulating gene expression related to inflammation and immune response when applied to treat pulmonary fibrosis. A study suggests that 5-aza-2′-deoxycytidine can induce human Tenon’s capsule fibroblasts differentiation and fibrosis by up-regulating TGF-β type I receptor (Fu et al., 2017). Moreover, there are also some reports showing interstitial and alveolar fibrosis (Adams et al., 2005), pneumonitis (Hayashi et al., 2012), and acute lung injury (Alyamany et al., 2024) in MDS patients after treated with azacitidine. If azacitidine induced lung toxicity occurs, it can be fatal, especially if not identified and treated in a timely manner (Alyamany et al., 2024). It is clear that less toxic inhibitors of DNA methylation are needed for clinical use.

## 4 Conclusion and perspective

IPF seriously endangers human health and life, but the pathogenesis of the condition remains unclear, and a dearth of effective pharmaceutical interventions persists. Therefore, it is very important to provide a theoretical basis for finding new methods of prevention and treatment of pulmonary fibrosis. In recent years, there has been a growing recognition of the close association between epigenetic alterations and lung diseases, with a significant impact on their pathogenesis, particularly through DNA methylation modifications, which have continuously developed and matured in epigenetics and has become a reliable biomarker. DNA methylation alternation is a dynamic and reversible process and is considered an effective therapeutic intervention. Many studies have also shown that DNA methylation and demethylation alternations play a certain role in IPF pathogenesis, such as abnormal methylation patterns (hypermethylation and hypomethylation), DNMTs and TET proteins, but the mechanisms of their actions in IPF are not particularly clear. Additional investigations into the roles and mechanisms of DNA methylation and demethylation in pulmonary fibrosis are anticipated to yield novel strategies for IPF prevention and treatment.
